# Supplementary figures and images for: Effect of conservation farming and biochar addition on soil organic carbon quality, nitrogen mineralization, and crop productivity in a light textured Acrisol in the sub-humid tropics
Source: PLoS One. 2020 Feb 6;15(2):e0228717. doi: 10.1371/journal.pone.0228717 (PMC7004324; doi:10.1371/journal.pone.0228717)

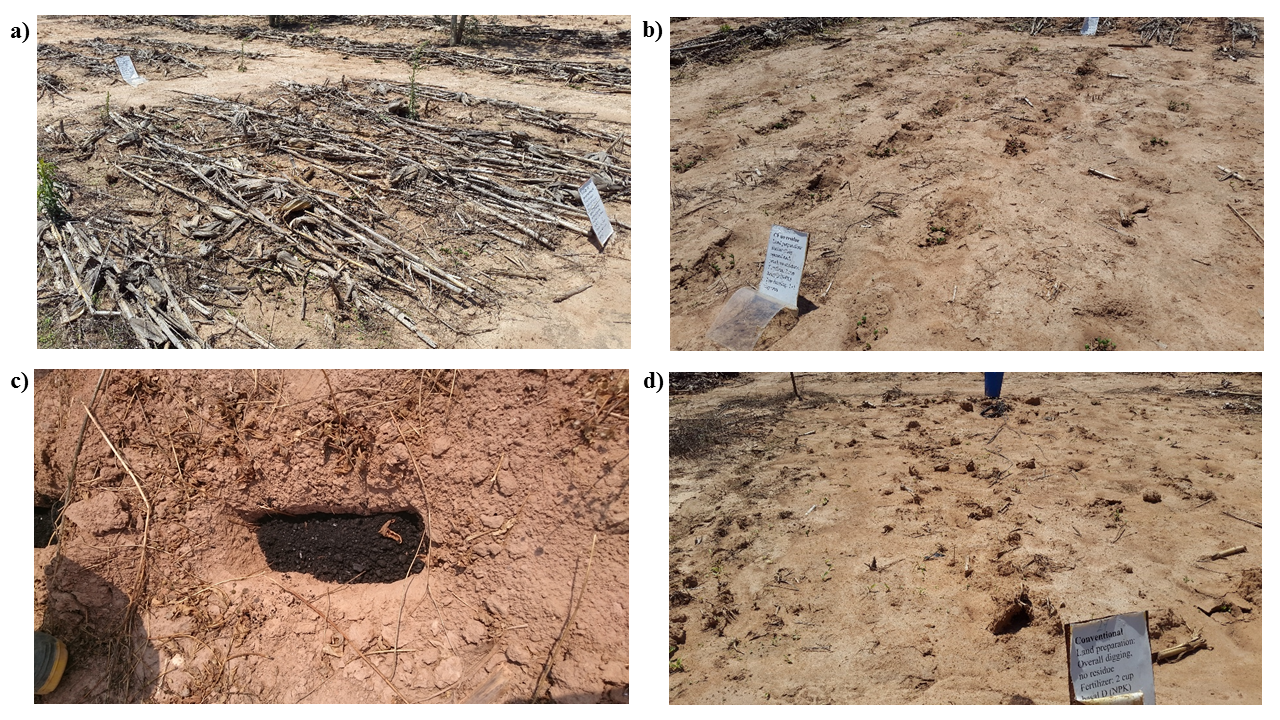

Supplement: S1 Fig — Soil regimes: a) CF-NORM: residue retention, permanent basins, b) CF-NO-RES: no crop residues without residue retention, c) CF-BC: addition of pigeon pea biochar inside basins and d) CONV: full tillage at a depth of 20 cm and no residue retention. (TIF) [file pone.0228717.s001.tif]

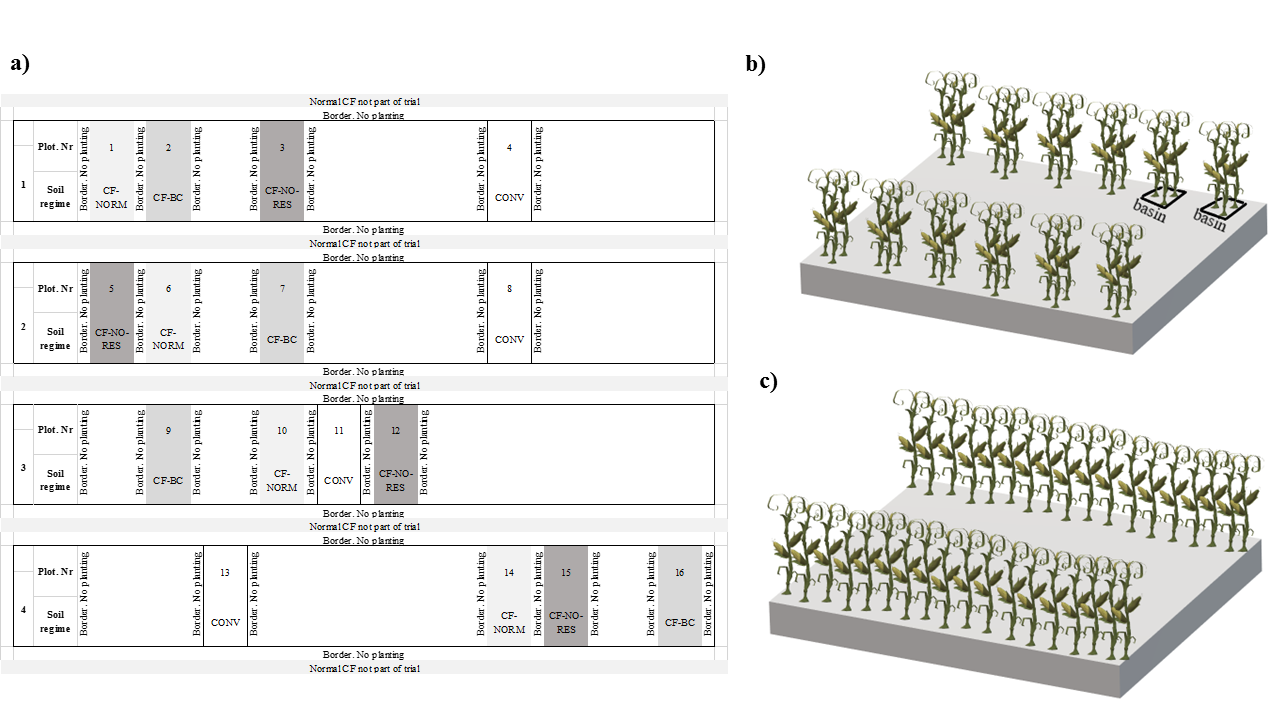

Supplement: S2 Fig — a) Experimental setup. The four soil regimes were randomly distributed in 4 blocks. Each plot was about 20 m2 and consisted of 4 rows of six basins in all the Conservation Farming (CF) treatments and 4 rows of plants in conventional (CONV) plots. b) CF plots. Each row consisted of 6 planting basins with 3 plants of maize. c) CONV plots. Each row had 18 plants. (TIF) [file pone.0228717.s002.tif]

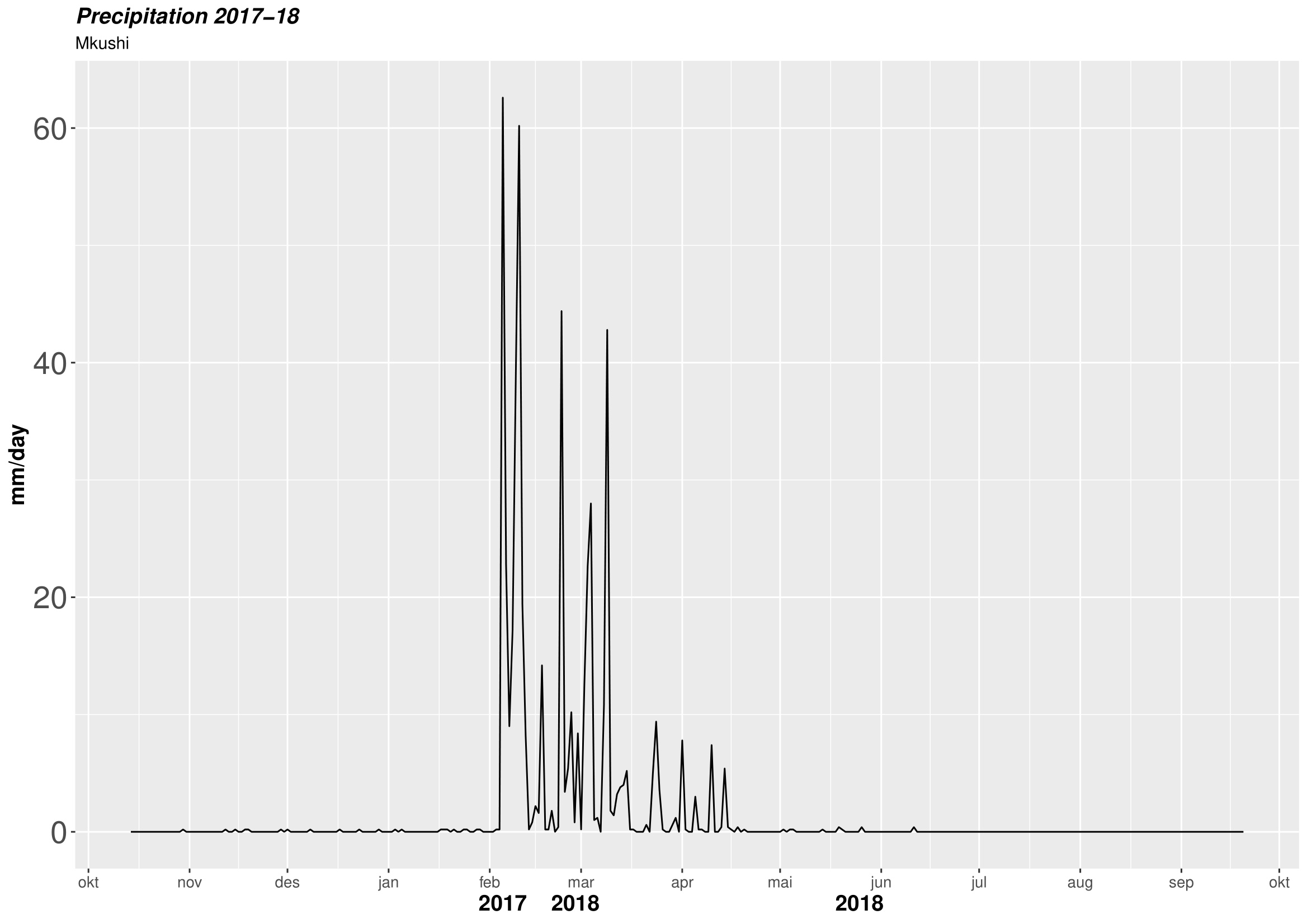

Supplement: S3 Fig — Precipitation from November 2017 to January 2018 was unusually low. The effect of the dry-spell on Zambia’s maize harvest was mentioned in the press: https://www.bloomberg.com/news/articles/2018-05-04/dry-spell-slashes-zambian-corn-production-by-34-in-2017-18 (TIFF) [file pone.0228717.s003.tiff]
