# Supplementary material for: Effect of conservation farming and biochar addition on soil organic carbon quality, nitrogen mineralization, and crop productivity in a light textured Acrisol in the sub-humid tropics
Source: PLoS One. 2020 Feb 6;15(2):e0228717. doi: 10.1371/journal.pone.0228717 (PMC7004324; doi:10.1371/journal.pone.0228717)
Supplement: S1 Table — (DOCX) [file pone.0228717.s008.docx]

**S1** **Tab.** Soil pH inside and outside basins in 2016 and 2018.

| **Soil pH** | **2016** | **2018** |
| --- | --- | --- |
| **Inside basins** |  |  |
| CF-BC | 6.5 (0.10) | 4.5 (0.04) |
| CF-NO-RES | - | 4.8 (0.13) |
| CF-NORM | 6.4 (0.10) | 4.5 (0.12) |
| CONV | 5.5 (0.12) | 4.6 (0.04) |
| **Outside basins** |  |  |
| CF-NO-RES | - | 4.9 (0.32) |
| CF-NORM | - | 4.5 (0.14) |
| CONV | - | 4.9 (0.12) |
